# Voluntary Aid Detachments

**Published:** 1910-09-17

**Authors:** 


### The Hospital

A Journal of

The Medical Sciences and Hospital Administration.

New Series. No. 188, Vol. VII. [No. 1260, Vol. XLVIII.] SATURDAY, SEPTEMBER 17, 1910.

### VOLUNTARY AID DETACHMENTS.

The War Office Advisory Committee for the Voluntary Aid Detachments, whose appointment we announced in our issue of August 6, has already held two meetings, and has put forward certain recommendations that have received the sanction of the War Office, and have now been officially announced as amendments in the scheme for the Organisation of Voluntary Aid in England and Wales. As they seem to us important, and to make for simplicity and economy, we feel it right to draw attention to them. Before, however, mentioning the now sanctioned alterations it is only right again to point out what we have already in previous articles emphasised, that the British Red Cross Society is still the only body specifically detailed in the War Office Scheme to undertake the formation of the Voluntary Aid Detachments whenever the County Associations do not themselves employ means for raising them; and, further, that the British Red Cross Society is the only body recommended by the War Office to the County Associations for the carrying out of this important work. We also note that the War Office very properly impresses on County Associations that, if they decide to work on other lines than those officially recommended, they must ensure that the standard of instruction is not lower than what is approved of by the War Office. In the new amendments issued there is no change in the appointment of the County Director, but the composition of both the Men's and Women's Detachments has been very much simplified. In the case of the men's the following is the sanctioned *personnel*: one commandant, one medical officer, one quartermaster, one pharmacist, four section leaders, and forty-eight men, divisible into four sections of twelve men each. For the Women's Detachments it is proposed that there should be one commandant, man or woman, and not necessarily a doctor, one quartermaster, man or woman, one trained nurse as lady superintendent, and twenty women, of whom four should be qualified as cooks. The concession that the commandant of a Women's Detachment may be a woman and the lady superintendent a trained nurse should prove helpful in the raising and working of the detachment, but the definition of a trained nurse has very properly been distinctly laid down and has a definite meaning. By it is understood a nurse who has completed a three years' course of training in a general hospital having a nurse-training school attached, and who, having qualified in the examinations of the institution, has received a certificate to this effect.

As regards the First-aid certificates that must be produced, with certain exceptions, by members of the Voluntary Aid Detachments, it has been decided that members need not have these on joining, but that they must produce them within twelve months of enrolment. Further, the War Office has enlarged the scope of certificate-granting bodies, recognising the certificate held by all ex-R.A.M.C. men, whether Regulars, Volunteers, or Territorials, and those given by the St. John's and St. Andrew's Ambulance Associations, by the London County Council, the National Health Society, the National Fire Brigades Union, and the Provincial County Councils, where the course of instruction has been given and the examination carried out by a duly qualified medical practitioner, it being, however, laid down that the instructor cannot be the examiner.

Another very important change that has been sanctioned is that the courses of instruction in home nursing may be given by trained nurses. This should prove of great assistance in country districts where the services of a medical man as lecturer might be difficult to obtain, but the concession is safeguarded by the fact that no "Home-nursing" certificate will be accepted unless the examination has been conducted by a medical practitioner or matron of a training school, either of whom must be distinct from the instructors of the class.

The above modifications in the regulations for the Voluntary Aid Detachments, together with revised forms for making the returns connected with them, constitute the chief amendments sanctioned by the War Office in their Scheme for the Organisation of Voluntary Aid in England and Wales. They do not apply to Scotland, but it is understood that the Scottish Red Cross Executive look favourably on them, and will adopt those that are not already in force, for some of these amendments formed part of the original scheme for Scotland.

The new concessions should prove of very great assistance in the establishment of the Voluntary Aid Detachments. For instance, the provision that allows of the division of a Men's Detachment into four sections should be a great convenience in rural districts, where a detachment may, if necessary, be made up of four sections in four adjoining villages. Another excellent and helpful arrangement is that certain candidates for the Men's Detachments may be admitted without First-aid Certificates, and that twelve months is allowed after enrolment for obtaining a certificate by those who require one.

